# Environmental Factors Determining the Accumulation of Metals: Cu, Zn, Mn and Fe in Tissues of *Taraxacum* sp. sect. *Taraxacum*

**DOI:** 10.1007/s00128-018-2356-y

**Published:** 2018-05-19

**Authors:** Elżbieta Królak, Jolanta Marciniuk, Katarzyna Popijantus, Paulina Wasilczuk, Zbigniew Kasprzykowski

**Affiliations:** Institute of Biology, Siedlce University of Natural Sciences and Humanities, Prusa 12, 08-110 Siedlce, Poland

**Keywords:** Heavy metals, Dandelion, Soil, Biological concentration factor, Translocation factor

## Abstract

The genus *Taraxacum* is used in the assessment of soil contamination with heavy metals. There are relatively few studies using sections or species representing this genus. The presented research was conducted in Poland on two habitats, varied in terms of nutrients and metals content. The content of selected metals in leaves and roots of *Taraxacum* sect. *Taraxacum* was determined. It was found that in the conditions of increased content of metals in the soil, the analysed species representing sect. *Taraxacum* accumulate higher amounts of metals in their leaves and roots. Factors of translocation of selected metals from roots to leaves of *Taraxacum* species, representing the *Taraxacum* section, are affected by i.a. soil reaction and the content of Corg, Ntot. in the soil. No influence of soil properties on metal biological concentration factor was observed.

The plant commonly used in environmental quality assessment is *Taraxacum* sp. The genus *Taraxacum* is one of the largest and taxonomically most diverse apomictic complexes. It is represented by approximately 60 sections that comprise about 2500 species together (Martinez et al. 2015).

The typical section *Taraxacum* sect. *Taraxacum* (Kirschner and Štěpánek 2011) is the most common and the largest group (i.e. represented by the largest number of species), more often referred to as *Taraxacum* sect. *Ruderalia*. The *Taraxacum* section includes mainly apomictic polyploids with a very wide indigenous range, covering nearly the whole of Europe, Western Asia and North Africa (Marciniuk et al. 2010), from where they have spread to all continents, except Antarctica (Quiroz et al. 2011; Srivastav et al. 2015). Species of the *Taraxacum* section are relatively large perennials with a strong taproot system and wide, usually lobed leaves arranged in a rosette. Large yellow flower heads are born singly on the tops of stems. The outer bracts of the involucre without spurs, usually with no rim or narrow rim, arranged in various ways—mostly drooping, reflexed or horizontally arranged. *Taraxacum* sp. is dominant in anthropogenic communities and its morphological features allow for its identification to the species. It is identified by e.g. the shape of its juvenile leaves which are collected during the flowering phase (Ooesterveld 1996).

The widespread geographical distribution of the genus *Taraxacum* and the section *Taraxacum*, the long life cycle, the ease of adaptation to each type of substrate as well as easy identification at the genus and typical section level make *Taraxacum officinale* (usually defined more broadly than the *Taraxacum* section) a good bioindicator in environmental quality assessment. Results of research carried out with the use of dandelions collected in anthropogenic habitats are often used in the identification of soil contamination e.g. with heavy metals such as Zn, Cu, Mn and Fe. These metals play significant metabolic functions in the human body and they are often studied in herbal plants, including tissues of *Taraxacum* (Bini et al. 2012; Cook et al. 1994; Čurlík et al. 2016; Kabata-Pendias and Dudka 1991; Królak 2003). Due to difficulties in the identification of dandelion species, most studies are conducted within the genus. Species representing the section *Taraxacum*, occurring in Poland in two different and heterogeneous locations, Siedlce and Zakopane, were selected for the purpose of this study. The following research hypotheses were adopted in the study: (I) habitats differ in the content of heavy metals in the soil, (II) plants representing the *Taraxacum* section occurring at chemically diversified habitats accumulate varied amounts of different heavy metals in their roots and leaves, (III) accumulation of metals in roots and leaves of the studied species from the section *Taraxacum* is determined by certain soil properties.

## Materials and Methods

Samples were collected in 2015 at two locations in Poland: in southern Poland (town: Zakopane, 49°17′N, 19°57′E, 819 m ASL) and in eastern Poland (town: Siedlce, 52°10′N and 22°17′E, 150 m ASL) in the phase of the plant intensive flowering (May). Zakopane is situated in a mountain valley (Zakopane Valley) at the foot of the Tatra Mountains. It is a tourist city with a population of about 27,000. Siedlce is located in the mesoregion of Siedlce Upland. It is an industrial and service town with a population of about 76,000. A total of 18 sites were sampled in Zakopane and 19 sites in Siedlce. In both towns, samples were taken from similar synanthropis habitats, from unmowed lawns and green spots located close to transport routes.

Soil samples and *Taraxacum* species representing the *Taraxacum* section were collected at each study site. For further research leaves rosettes and stems were used. At the time of samples collection, the leaves rosettes were at similar growth stage so they did not differ physiologically. Due to different growth stages of flower stalks and inflorescences, they were not taken into account in the research. All the soils samples were taken from anthropogenic sites. Approximately 1 kg soil samples were collected from a depth of 20 cm at each site, using Egner’s sampling stick. Along with soil sampling, 3–5 specimens representing species from *Taraxacum* sect. *Taraxacum* were collected. Samples collected in the field were properly preserved and transported to a laboratory.

In the laboratory the following parameters were determined in the soil samples: pH, Corg, Ntot., Ptot., the content of Cu, Zn, Mn and Fe. Soil pH was measured in 1 M KCl (1:2.5 ratio) using a glass electrode pH-meter, and organic carbon was determined with the Tiurin method (Ostrowska et al. 1991). In order to determine the content of Ntot. and Ptot. in the soil, soil samples (0.3000 g) were mineralized in Kjeldahl flasks, using 95% H_2_SO_4_ and 30% H_2_O_2_ (3:1, v/v). The total soil nitrogen content was determined with the phenyl hypochlorite method (Solórzano 1969), and total phosphorus with the molybdenum blue method (Standard Methods 1999).

To determine the content of metals, 1 g soil subsamples were pre-mineralized in a muffle furnace at 420°C and then mineralized in the mixture of HNO_3_ (68%) and H_2_O_2_ (30%), concentrated nitric acid enriched with hydrogen oxide (3:2, v/v), in a microwave mineralizer. After mineralization, the samples were transferred quantitatively to 50 cm^3^ flasks. In addition to soil, the content of metals was also determined in leaves and roots of dandelions. To this end, dandelion leaves and roots were washed thoroughly with redistilled water and air dried, and then dried at 60°C. The samples were homogenized and 5 g subsamples of dry mass of leaves and roots were prepared for analysis. Mineralization of dandelion leaves and roots was carried out in the same way as mineralization of soil samples. The weight of leaf and root samples after mineralization was 1.000 g. The content of copper, zinc, manganese and magnesium in soil, leaves and roots were determined with the use of the AAS technique (an apparatus manufactured by Carl Zeiss Jena), using acetylene-air flame for analysis. Standard solutions within the following concentration ranges (µg/mL): Cu: 0.2–3.0, Zn: 0.3–3.6, Mn: 0.5–5.0, Fe: 0.6–6.0 were used for the determination of individual metals. In the case of high metal content in the samples, exceeding the concentration range of standard solutions, the examined samples were diluted. Each analysis was repeated twice. Simultaneously, the analyses for the blank test were carried out. Polish Virginia Tobacco Leaves (INCP–PVTL–6) prepared and certified at the Institute of Nuclear Chemistry and Technology (Warsaw, Poland) were used as reference material in the analysis of metal ion concentration.

The results of the analysis of selected soil chemical parameters and the content of metals in dandelion leaves and roots were calculated per 1 g d.m. of a given analysed sample. The normality of data was tested using the Shapiro–Wilk test. To compare the values of the measured parameters in the samples collected in Zakopane and Siedlce, the Mann–Whitney test was used. The statistical analyses were performed using STATISTICA software (ver. 12).

Based on the ratio: the concentration of a metal in the root and the concentration of a metal in the soil, values of metal biological concentration factors (BCF) were calculated, while based on the ratio: the concentration of a metal in leaves and the concentration of a metal in the root, values of metal translocation factors (TF) were calculated (Čurlík et al. 2016; Malik et al. 2010). Canonical correspondence analysis (CCA) was performed to determine the effect of selected chemical indices on the cumulation and translocation factors.

## Results and Discussion

Soil samples collected at the same sites as dandelions were characterised by neutral (pH 6.6–7.2) or slightly alkaline reaction (pH > 7.2). The content of organic carbon and nitrogen in the samples varied in a wide range, from 0.300% to 9.610% C and from 0.264 to 6.370 mg N/g dm, respectively. Based on the determined values of C and N, the C/N ratio in the soil was calculated, which ranged from 5.47 to 85.56. The content of phosphorus in the soil was also characterised by a wide range: from 0.264 mg/g dm to 1.543 mg P/g dm. Soil samples from Zakopane were characterised by a higher content of nitrogen (Z = 3.87, *p* < 0.001), phosphorus (Z = 3.48, *p* < 0.001) and carbon (Z = 3.28, *p* = 0.001) compared to samples collected in Siedlce (Table 1).

**Table 1:** Selected soil chemical parameters at the study sites in Zakopane and Siedlce

Parameter	Unit	Zakopane				Siedlce			
		Arithmetic mean ± SD	Min.	Max	Median	Arithmetic mean ± SD	Min	Max	Median
Acidity	pH		6.62	7.51	6.93		6.64	7.65	7.20
N	mg/g	2.840 ± 1.380^a^	1.100	6.370	2.605	1.993 ± 1.495^b^	0.264	6.370	0.757
P		0.759 ± 0.329^a^	0.343	1.543	0.707	0.600 ± 0.302^b^	0.264	1.348	0.383
C	%	5.123 ± 2.222^a^	1.686	9.610	5.275	3.717 ± 2.608^b^	0.300	9.210	1.210
C/N		18.76 ± 5.80	13.28	35.37	17.18	21.14 ± 15.56	5.47	85.56	15.98

^a,b^Statistically significant differences between the values of studied parameters

**Fig. 1:**
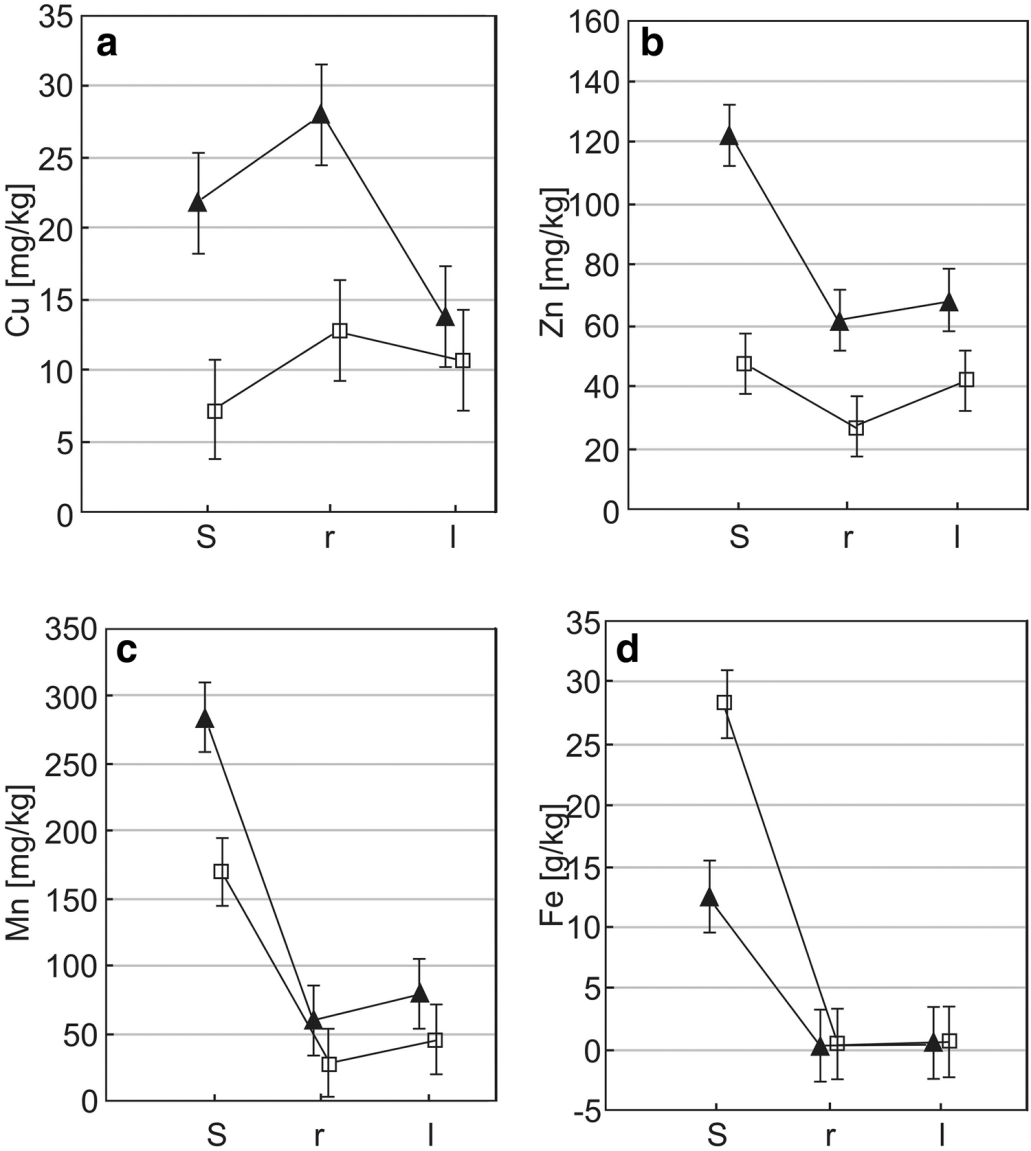
The content of metals: copper (**a**), zinc (**b**), manganese (**c**) and iron (**d**) in the soil (s), roots (r) and leaves (l) of *Taraxacum* sp. section *Taraxacum* in urban areas of Siedlce (□) and Zakopane (▲)

Analysis of Cu, Zn, Mn and Fe content (Fig. 1a–d) in the soil samples collected at the sites in Zakopane and Siedlce as well as in the roots and leaves of the analysed species representing the *Taraxacum* section showed statistically significant differences (*p* < 0.001, n = 37) between the locations. Higher values of Cu (Z = 4.66), Zn (Z = 5.06) and Mn (Z = 3.63) were determined in soil samples collected in Zakopane compared to soil samples from Siedlce. On the other hand, the content of Fe in soil samples from Siedlce was more than two times higher compared to samples from Zakopane (Z = 4.39). The concentration of Cu (Z = 3.18,), Zn (Z = 3.41) and Mn (Z = 3.17) in leaves of dandelions collected in Zakopane were significantly higher (*p* < 0.001, n = 37) compared to the content of these metals in leaves of plants collected in Siedlce. Similarly as in the case of leaves, significant differences (*p* < 0.001) in the content of these metals (Cu–Z = 4.15, Zn–4.66, Mn–Z = 3.84) were also determined in the roots of dandelions representing the *Taraxacum* section. The content of Fe was higher in dandelions collected in Siedlce compared to Zakopane, both in leaves (Z = 3.63, n = 37, *p* < 0.001) and roots (Z = 2.08, *p* = 0.037).

Statistical analysis showed statistically significant (*p* < 0.001) correlations between the content of Cu in the soil and the content of Cu in leaves (R_s_ = 0.555) and roots of dandelions (R_s_ = 0.555), the content of Zn in the soil and the content of Zn in leaves (R_s_ = 0.649) and roots (R_s_ = 0.727), as well as the content of Mn in the soil and in leaves (R_s_ = 0.366, *p* < 0.05) and the content of Mn in the soil and roots (R_s_ = 0.411, *p* < 0.05). Statistically significant correlations were also determined between the content of Fe in the soil and content of Fe in the leaves (R_s_ = 0.572, *p* < 0.001) and the roots (R_s_ = 0.505, *p* < 0.01) of dandelions.

The highest values of biological concentration factor (BCF) were recorded for Cu, i.e. above 1 at both locations. For other metals, values of BCF were below 1 (Table 2). Values of cumulation factors did not differ significantly between the locations. Values of translocation factors (TF) were above 1 for Zn, Mn and Fe, and below 1 for Cu. Differences in the values of translocation factors (TF) between the locations were statistically significant in the case of Cu (Z = 3.99, *p* < 0.001), Zn (Z = 2.17, *p* = 0.028) and Fe (Z = 2.45, *p* = 0.013).

**Table 2:** Mean values of the biological concentration factor (BCF) and the transfer factor (TF) of metals in Zakopane and Siedlce

Metal	Zakopane		Siedlce	
	BCF	TF	BCF	TF
Cu	1.67 ± 1.08^a^	0.55 ± 0.17^b^	2.24 ± 1.61^a^	0.97 ± 0.26^c^
Zn	0.54 ± 0.22^a^	1.15 ± 0.32^b^	0.78 ± 0.28^a^	1.92 ± 0.88^c^
Mn	0.26 ± 0.21^a^	1.39 ± 0.58^b^	0.16 ± 0.18^a^	2.08 ± 2.31^b^
Fe	0.023 ± 0.007^a^	1.64 ± 2.84^b^	0.021 ± 0.007^a^	1.88 ± 1.28^c^

^a,b,c^Statistically significant differences between the values of studied parameters

**Fig. 2:**
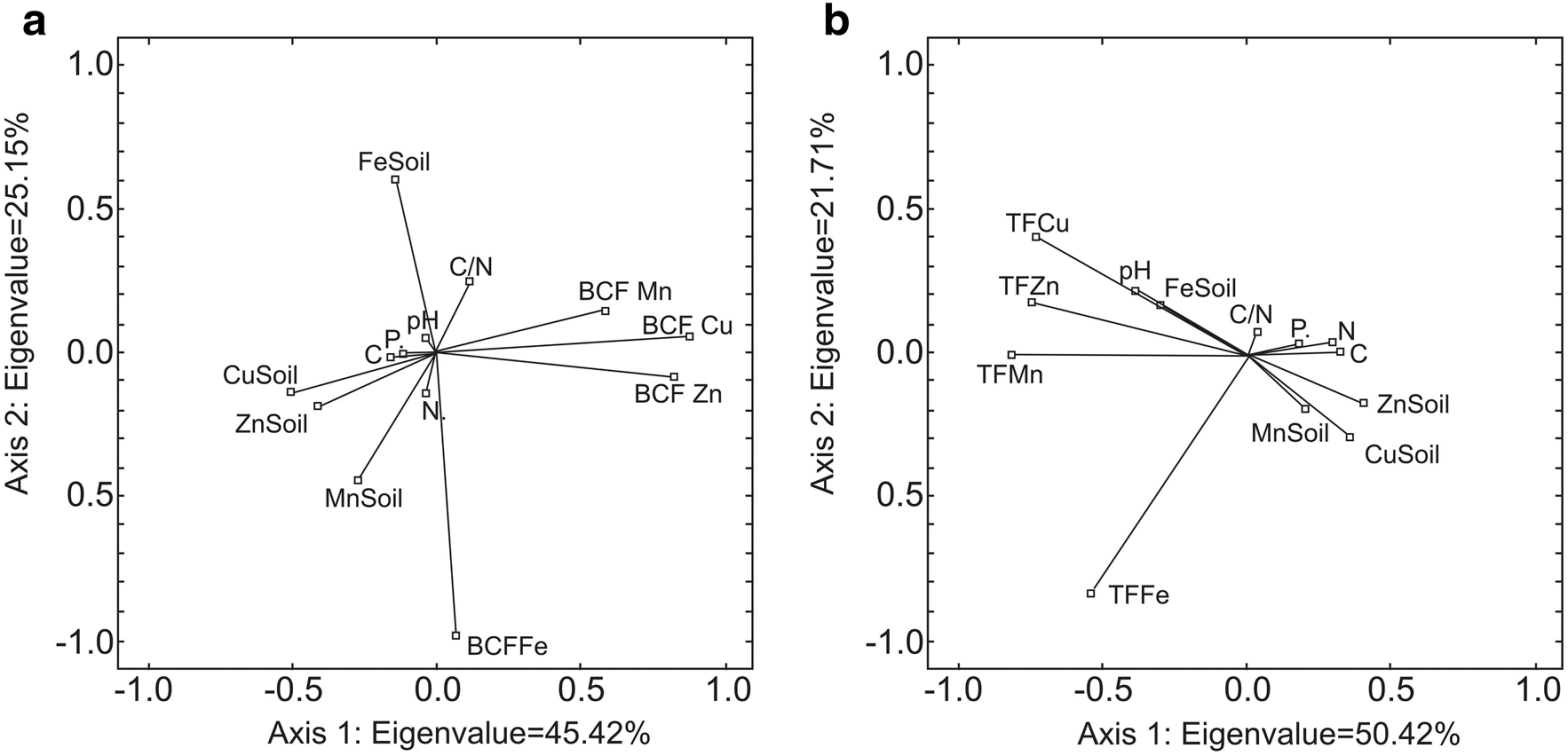
Results of CCA analysis describing the relationship between BCF values (**a**) and TF values (**b**) and selected soil properties

The CCA analysis showed that the analysed soil parameters did not significantly affect the values of metal biological concentration factor (BCF). On the other hand, a significant effect of nitrogen (R_s_ = − 0.42) and carbon (R_s_ = − 0.40) content on TF values of Cu was observed, the effect of soil pH (R_s_ = 0.36) on TF values of Zn and the effect of nitrogen content in the soil (R_s_ = 0.36) on TF values of Fe. The two axes (Fig. 2a, b) accounted for above 70% of the predictors variability.

The studied *Taraxacum* section occurred in habitats differing in the content of organic carbon, nitrogen and phosphorus. According to the available literature data (Bini et al. 2012; Stewart-Wade et al. 2002), the *Taraxacum* genus can grow in a wide range of soil types and adapt to a wide range of light and shade intensity. It also occurs in soils characterised by a wide range of pH values (from 4.5 to 7.8). In our research, soil samples collected at the same sites as sect. *Taraxacum* species were characterised by neutral or slightly alkaline reaction. Significantly higher differences in the content of Cu, Zn, Mn were recorded in the surface layer of soil collected near Zakopane, compared to Siedlce. Whereas the higher content of Fe was determined in the soil from Siedlce compared to Zakopane. Differences in the content of metals in the soil of both locations may depend on the influx of anthropogenic contamination. An important anthropogenic source of heavy metals in the soil is dustfall, especially PM10 and PM2.5. It is worth noting that, compared to Siedlce, the annual average PM10 and PM2.5 concentration is higher in Zakopane where it often exceeds admissible levels set by the Polish Ministry of Environment at 40 and 25 µg/m^3^, respectively (Report 2017a, b).

Zn and Cu present in the atmosphere come from fine fractions of particulate matter released during energy production processes (Kowol et al. 2011; Krčmová et al. 2009; Manousakas et al. 2013). Also linear emission connected with traffic may result in the increased content of heavy metals in soil. Many studies (Cook et al. 1994; Degórska 2013; Ewen et al. 2009; Giacomino et al. 2016; Ianovici 2016; Korzeniowska and Panek 2010; Krčmová et al. 2009) have reported that urban dust collected in streets is contaminated with trace metals, for example: zinc (Zn) and copper (Cu). According to the reports of General Directorate of National Roads and Motorways (https://www.gddkia.gov.pl), traffic intensity on roads located in close vicinity of sampling sites is higher in Zakopane than in Siedlce (by more than 7500 vehicles a day). What is more, due to its topography, Zakopane often gets foggy and air frequently remains stagnant in the town which contributes to further air pollution. Air ventilation is much better in Siedlce which is a result of the town location.

The concentration of Zn was above 100 mg/kg in more than 70% of soil samples collected in Zakopane and the content of Cu exceeded the value of 25 mg Cu/kg in more than 20% of samples from Zakopane. The value of 100 mg Zn/kg was not exceeded in any of the analysed soil samples collected in Siedlce and only one sample from Siedlce showed the content of Cu above 25 mg/kg; it is assumed that these values reflect the natural content of metals in soils of Poland (Kabata-Pendias et al. 1995).

The concentrations of heavy metals in *T. officinale* species depend on their concentrations in the soil (e.g. Cook et al. 1994; Keane et al. 2001; Savinov et al. 2007) and this was confirmed by our research. The higher the content of Cu, Zn, Mn in the soil, the higher their content in leaves and roots of plants collected in Zakopane compared to Siedlce. It is worth noting that concentrations of all of the studied metals in leaves collected in Zakopane were above background values (Cu—11.2, Zn—49.6, Mn—59.7, Fe—184 mg/kg d.w.) estimated for southern Poland by Kabata-Pendias and Dudka (1991). In the case of leaves samples taken in Siedlce, mean values of copper and manganese did not exceed background values estimated for northern Poland to 11.1 mg Cu/kg d.w. and 51.4 mg Mn/kg d.w. Zinc concentration in dandelion leaves was comparable to its background value − 41.3 mg/kg d.w.

The statistically significant correlation between the concentration of the analysed metals in the soil and their content in leaves and roots of plants corroborates the literature data (Bini et al. 2012; Collier et al. 2010; Cook et al. 1994; Čurlík et al. 2016; Królak 2003) on the usefulness of species from the *Taraxacum* genus, including the *Taraxacum* section, in bioindication studies. It should also be noted that the studied species accumulated higher amounts of Zn, Mn and Fe in leaves compared to roots, while more Cu was accumulated in roots than in leaves. Similar results on the translocation of Cu and Zn in the plant were obtained by Bini et al. (2012), Królak (2003), and of Mn—by Gjorgieva et al. (2011). Higher values of translocation factors (TF) compared to biological concentration factor (BCF) for Zn, Mn and Fe suggest that plants are capable of relocating these metals from roots to aerial parts. Copper is much less susceptible to relocation from roots to leaves, which is evidenced by smaller values of the translocation factor compared to the biological concentration factor. Lack of statistically significant differences in BCF values calculated for both locations may indicate that the factor is not a useful tool in bioindication studies. Translocation factor (TF) seems to be a better indicator of environmental pollution which is confirmed by statistically significant differences in TF values noted for Cu, Zn and Fe in Zakopane and Siedlce. Cook et al. (1994) claim that the plant/soil concentration ratio of metals depends on the content of metals in soil. The values of this ratio are lower the higher concentrations of the studied metals in soil which is reflected in our research in the values calculated for Cu and Zn.

Assimilation of metals by plants is determined by certain soil properties, i.a. the content of organic matter, soil reaction (Bini et al. 2012; Mikulka et al. 2009; Niesiobędzka 2012). The availability of certain metals for plants, e.g. Cu, Zn, Fe increases along with increasing soil acidity (Kabata-Pendias and Pendias 1993; Niesiobędzka 2012) and in the case of manganese it also depends on redox conditions. In the acid environment, manganese is absorbed by plants in the cationic form, while in the alkaline environment (pH > 8)—mainly in the form of anionic forms. Other metals, depending on pH of soil, are present in the soil solution in the form of simple cations, or these cations form chelate complexes that are absorbed by plants (Kabata-Pendias and Pendias 1993). With the soil pH measured in our study in the range of 6.62–7.65, the transfer of Fe and Mn is limited, which is evidenced by low values of biological concentration factor (BCF) for these metals in both locations.

Soil properties are listed among the main factors determining the cumulation or translocation of metals in the plants (Malik et al. 2010). The research showed that the values of Cu, Zn, Mn and Fe biological concentration factors do not depend on such properties of soil as: (pH of soil, the content of Ntot., Ptot., Corg.). On the other hand, soil reaction affected the TF values of Zn, while the content of Ntot. and Corg. in the soil determined the TF values of copper. No significant effect of soil properties on the TF values of Mn was noted. Of the analysed metals, copper is most strongly bound by soil organic matter. Organic matter has a tendency to form complexes with heavy metals and, consequently, it may contribute to e.g. increased mobility of some metals (Vega et al. 2004). Some authors (Bini et al. 2012; Ianovici 2016; Keane et al. 2001) emphasize that factors affecting the accumulation of metals in dandelion leaves are complex and often other environmental factors, in addition to the metals content in the soil, are crucial, e.g. the content of nutrients in the environment (Bini et al. 2012; Wielgolaski 2001).

The usefulness of species representing the genus *Taraxacum* sp. in the bioindication methods has not yet been fully researched. There are no data on the accumulation of metals in individual species representing the genus *Taraxacum* (Kováčik et al. 2016). In order to better correlate the accumulation of metals in selected *Taraxacum* sections or species, further research in this field is necessary.

## References

[CR1] Bini C, Wahsha M, Fontana S, Maleci L (2012). Effects of heavy metals on morphological characteristics of *Taraxacum officinale* web growing on mine soils in NE Italy. J Geochem Explor.

[CR100] Collier MH, Keane B, Rogstad SH (2010). Productivity differences between dandelion (*Taraxacym officinale*; *Asteraceae*) clones from pollution impacted versus non-impacted soils. Plant Soil.

[CR2] Cook CM, Sgardelis SP, Pantis JD, Lanaras T (1994). Concentrations of Pb, Zn, and Cu in *Taraxacum* spp. in relation to urban pollution. Bull Environ Contam Toxicol.

[CR3] Čurlík J, Kolesár M, Ďurža O, Hiller E (2016). Dandelion (*Taraxacum officinale*) and agrimony (*Agrimonia eupatoria*) as indicators of geogenic contamination of flysch soils in eastern Slovakia. Arch Environ Contam Toxicol.

[CR4] Degórska A (2013). An assessment of urban habitat contamination with selected heavy metals within the city of Katowice using the common dandelion (*Taraxacum officinale* Web.) as a bioindicator. Environ Socio-Econ Stud.

[CR5] Ewen C, Anagnostopoulou MA, Ward NI (2009). Monitoring of heavy metal leveles in roadsite dusts of Thessaloniki, Greece in relation to motor vehicle traffic density and flow. Environ Monit Assess.

[CR6] Giacomino A, Malandrino M, Colombo ML, Miaglia S, Maimone P, Blancato S, Conca S, Abollino E (2016). Metal content in dandelion (*Taraxacum officinale*) leaves: influence of vehicular traffic and safety upon consumption as food. J Chem.

[CR7] Gjorgieva D, Kadifkova-Panovska T, Bačeva K, Stafilov T (2011). Assessment of heavy metal pollution in Republic of Macedonia using a plant assay. Arch Environ Contam Toxicol.

[CR8] Ianovici N (2016). *Taraxacum officinale* (*Asteraceae*) in the urban environment: seasonal fluctuations of plant traits and their relationship with meteorological factors. Acta Agrobot.

[CR9] Kabata-Pendias A, Dudka S (1991). Trace metal contents of *Taraxacum officinale* (dandelion) as a convenient environmental indicator. Environ Geochem Health.

[CR10] Kabata-Pendias A, Pendias H (1993). Biogeochemistry of trace elements.

[CR11] Kabata-Pendias A, Piotrowska M, Motowicka-Terelak T, Maliszewska-Kordybach B, Filipiak K, Krakowiak A, Pietruch C (1995). Basics of chemical assessment of soil pollution. Heavy metals, sulfur and PAHs.

[CR12] Keane B, Collier MH, Shann JR, Rogstad SH (2001). Metal content of dandelion (*Taraxacum officinale*) leaves in relation to soil contamination and airborne particulate matter. Sci Total Environ.

[CR13] Kirschner J, Štěpánek J (2011). Typification of *Leontodon taraxacum* L. (= *Taraxacum officinale* F.H. Wigg.) and the generic name *Taraxacum*: a review and a new typification proposal. Taxon.

[CR14] Korzeniowska J, Panek E (2010). Heavy metal (Cd, Cr, Cu, Ni, Pb, Zn) concentrations in Norway spruce *Picea abies* L. along the roads of various traffic density in the Podhale region, southern Poland. Geomat Environ Eng.

[CR15] Kováčik J, Dudáš M, Hedbavny J, Mártonfi P (2016). Dandelion *Taraxacum linearisquameum* does not reflect soil metal content in urban localities. Environ Pollut.

[CR16] Kowol J, Kwapuliński J, Brodziak-Dopierala B, Paukszto A, Bogunia M, Rochel R, Ahnert B (2011). Influence of a transboundary emission on bioavailability of metals of stinging nettle from soil. Pol J Environ Stud.

[CR17] Krčmová K, Robertson D, Cvečková V, Rapant S (2009). Road-deposited sediment, soil, and precipitation (RDS) in Bratislava, Slovakia: compositional and spatial assessment of contamination. J Soil Sediment.

[CR18] Królak E (2003). Accumulation of Zn, Cu, Pb and Cd by Dandelion (*Taraxacum officinale* Web.) in environments with various degrees of metallic contamination. Pol J Environ Stud.

[CR19] Malik RN, Husain SH, Nazir I (2010). Heavy metals contamination in soil and wild plant species from industrial area of Islamabad, Pakistan. Pak J Bot.

[CR20] Manousakas M, Eleftheriadis K, Papaefthymiou H (2013). Characterization of PM10 sources and ambient air concentration levels at Megalopolis City (Southern Greece) located in the vicinity of lignite-fired plants. Aerosol Air Qual Res.

[CR21] Marciniuk P, Marciniuk J, Grużewska T, Głowacki Z (2010) The genus *Taraxacum* in Poland. General knowledge, collection and determination. Publisher of the University of Natural Sciences and Humanities in Siedlce, Monographs nr 119, p 113 (in Polish)

[CR22] Martinez M, Poirrier P, Chamy R, Prüfer D, Schulze-Gronover C, Jorquera L, Ruiz G (2015). *Taraxacum officinale* and related species—an ethnopharmacological review and its potential as a commercial medicinal plant. J Ethnopharmacol.

[CR23] Mikulka J, Korčáková M, Burešová V, Andr J (2009). Changes in weed species spectrum of perennial weeds on arable land, meadows and pastures. Plant Protect Sci.

[CR24] Niesiobędzka K (2012). Transfer of copper, lead and zinc in soil-grass ecosystem in aspect of soils properties, in Poland. Bull Environ Contam Toxicol.

[CR25] Ooesterveld P (1996). Phenological problems in the growth of dandelions. What is a representative dandelion?. Taraxacum Newslett.

[CR26] Ostrowska A, Gawliński S, Szczubiałka Z (1991). Methods of analysis and assessment of soil properties and plant.

[CR27] Quiroz CL, Cavieres LA, Pauchard A (2011). Assessing the importance of disturbance, site conditions, and the biotic barrier for dandelion invasion in an Alpine habitat. Biol Invasions.

[CR35] Report on the state of the environment in the małopolskie voivodship in 2016 (2017a) Provincial Inspectorate for Environmental Protection, Cracow (in Polish)

[CR36] Report on the state of the environment in the mazowieckie voivodship in 2016 (2017b) Provincial Inspectorate for Environmental Protection, Warsaw (in Polish)

[CR28] Savinov AB, Kurganova LN, Shekunov YI (2007). Lipid peroxidation rates in *Taraxacum officinale* Wigg. and *Vicia cracca* L. from biotopes with different levels of soil pollution with heavy metals. Russ J Ecol.

[CR29] Solórzano L (1969). Determination of ammonia in natural waters by the phenylhypochlorite method. Limnol Oceanogr.

[CR30] Srivastav M, Kumar A, Hussain T (2015). Diversity of angiospermic plants in Dhanaulti Region, Uttarakhand: an emerging tourist destination in Western Himalaya. Check List.

[CR31] Standard Methods for the Examination Water and Wastewater (1999) In: Clescerl LS, Greenberg AE, Eaton AD (eds), American Public Health Association, New York

[CR32] Stewart-Wade SM, Neumann S, Collins LL, Boland GJ (2002). The biology of Canadian weeds. 117. *Taraxacum officinale* G. H. Weber ex Wiggers. Can J Plant Sci.

[CR33] Vega FA, Covelo EF, Andrade ML, Marcet P (2004). Relationships between heavy metals content and soil properties in minesoils. Anal Chim Acta.

[CR34] Wielgolaski FE (2001). Phenological modifications in plants by various edaphic factors. Int J Biometeorol.

